# Neural Network Assisted Pathology Case Identification

**DOI:** 10.1016/j.jpi.2022.100008

**Published:** 2022-01-20

**Authors:** Jerome Cheng

**Affiliations:** Department of Pathology, University of Michigan, Ann Arbor, MI, USA

**Keywords:** Natural language processing, Word embedding, Neural network, Structured query language, Laboratory information system

## Abstract

**Background:**

Traditionally, cases for cohort selection and quality assurance purposes are identified through structured query language (SQL) searches matching specific keywords. Recently, several neural network-based natural language processing (NLP) pipelines have emerged as an accurate alternative/complementary method for case retrieval.

**Methods:**

The diagnosis section of 1000 pathology reports with the terms “colon” and “carcinoma” were retrieved from our laboratory information system through a SQL query. Each of the reports were labeled as either positive or negative, where cases are considered positive if the case was a primary adenocarcinoma of the colon. Negative cases comprised adenocarcinoma from other sites, metastatic adenocarcinomas, benign conditions, rectal cancers, and other cases that do not fit in the primary colonic adenocarcinoma category. The 1000 cases were randomly separated into training, validation, and holdout sets. A convolutional neural network (CNN) model built using Keras (a neural network library) was trained to identify positive cases, and the model was applied to the holdout set to predict the category for each case.

**Results:**

The CNN model classified 141 out of 149 primary colonic adenocarcinoma cases, and 43 out of 51 negative cases correctly, achieving an accuracy of 92% and area under the ROC curve (AUC) of 0.957.

**Conclusion:**

Trained convolutional neural network models by itself, or as an adjunct to keyword and pattern-based text extraction methods may be used to search for pathology cases of interest with high accuracy.

## Background

Pathology reports are commonly retrieved from a laboratory information system (LIS) through a structured query language (SQL) query. In some systems, the data are stored as unstructured free text, often requiring significant effort to transform the data into discrete format before it can be analyzed or used for machine learning purposes. Recent advances in natural language processing (NLP) techniques and the increasing availability of open-source tools have made it more convenient to process free-text data using word embedding methods and neural networks. Through word embedding, words can be converted into a series of numbers, which in turn can be used to train machine learning models.

Historically, Word2Vec played a major role in popularizing word embedding approaches, while achieving state-of-the-art performance during its time, and to this day, many of the more recent NLP approaches are based on word embeddings (e.g., fastText^1^ and BERT^2^). Word2Vec maps words to a multidimensional vector representation based on word proximity, encoding words with their contextual meaning in a numerical representation.^3^ Potential use cases for word embeddings include similarity searches (may be used to look for cases worded similarly to a particular report), case classification/categorization, and automated labeling of free-text pathology reports.^4^ There are a multitude of papers applying NLP techniques to free-text data (e.g., patient encounter notes in electronic medical records^5^ and radiology reports^6^) but there are currently few NLP publications related to the field of anatomic pathology. Currently, many studies center on using machine learning methods such as convolutional neural networks for image classification,^7^ segmentation,^8^ or stain normalization.^9^ Of note, many techniques used on images can also be applied to text-based data, such as convolutional neural networks, which was used in this study to classify free-text pathology reports.

## Methods

The diagnosis section of 1000 anatomic pathology reports were retrieved from our anatomic pathology LIS in CSV format through a SQL query using “colon” and “carcinoma” as keywords. Using LibreOffice, primary colonic adenocarcinoma cases were manually labeled as positive (coded as 1), while other types of cases were labeled as negative (coded as 0), which included metastatic adenocarcinoma and rectal adenocarcinoma cases. A total of 713 cases were labeled as positive (1), and 287 cases were labeled negative (0). The CSV file comprised two columns – the first column contained the label (0 or 1), and the second column contained the free-text report diagnosis. All cases were randomly assigned into training, validation, and holdout sets, with a 60/20/20 ratio. 425 positive cases were in the training set, 139 in the validation set, and 149 in the holdout set.

A neural network with embedding, 2 one-dimensional convolution, global max pooling, and dense layers was built using Keras (https://keras.io) on a system running Windows 10, TensorFlow 2.6(GPU version), Python 3.7 in an Anaconda environment. Computer hardware specifications included an Intel I9-9900KF CPU, 64 GB of RAM, and an Nvidia RTX 3090 GPU. Each individual report from all datasets was separated into individual words and each word was represented by a unique number using the keras.preprocessing.text.Tokenizer.texts_to_sequences function of Keras. Since convolutional neural networks work with data of uniform size, each sequence less than 200 elements in length was padded by zeroes using the keras.preprocessing.sequence.pad_sequences function. The neural network model was trained with a batch size of 64, input length of 200, vocabulary size of 1056 (derived from the total number of unique words), the RMSprop optimizer with its default settings, the binary cross-entropy loss function, using the training and validation sets. Model layers were altered and hyperparameters were fine-tuned until a validation accuracy of 90.5% was achieved using 0.5 as the positive cut-off, and the final model was applied on the holdout dataset. Optimal accuracy was achieved after the 11th epoch. The holdout set predictions and true labels were exported as a CSV file, and an ROC curve was generated using the IBM SPSS Statistics Version 28 statistical package.

Manual hyperparameter exploration included the kernel size and number of filters of the convolutional layers, number of units in the dense layer, maximum sequence input length, and the embedding dimension.

The following code segment written in Python and Keras details the final hyperparameters and model layers that were empirically derived (embedding dimension of 90, input length of 200):

from keras.models import Sequential

from keras import layers

model = Sequential()

model.add(layers.Embedding(vocabulary_size, 90, input_length=200))

model.add(layers.Conv1D(256,7, activation='relu'))

model.add(layers.Conv1D(512,9, activation='relu'))

model.add(layers.GlobalMaxPooling1D())

model.add(layers.Dense(256, activation='relu'))

model.add(layers.Dense(1, activation='sigmoid'))

## Results

**Figure 1:**
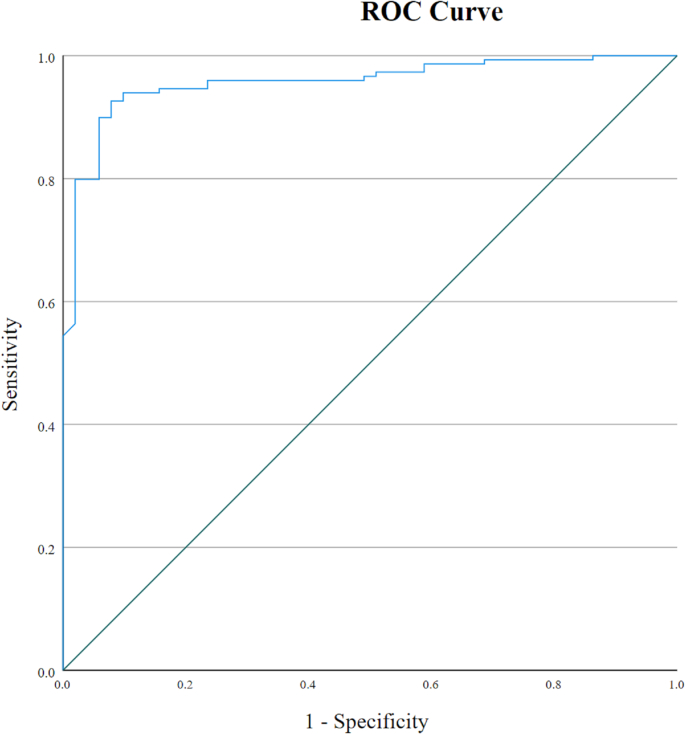
Receiver operating characteristic curve based on predictions made by the trained neural network on the holdout set, showing an area under the ROC curve of 0.957.

Since the training set is relatively small, it only needed a few seconds and 11 training epochs for the model to converge and achieve peak accuracy. Using 0.5 as the positive cut-off, the neural network model correctly classified 141 out of 149 primary colonic adenocarcinoma cases, and 43 out of 51 negative cases, achieving an accuracy of 92%. This is an improvement over the original SQL keyword search that attained an accuracy of 71.3% (713 out of 1000 cases). The plotted ROC Curve had an area under the curve (AUC) of 0.957 (Fig. 1).

## Discussion

Traditionally, case searches for cohort selection and quality assurance purposes involved looking for the presence of keywords or text patterns within pathology reports. Recently, there has been an emergence of various neural network-based NLP pipelines that are efficient and accurate in text classification, which can reduce the amount of manual effort given the proper use cases. Once NLP models have been trained on a set of labeled cases (e.g., benign vs. malignant), these can be used to categorize reports into the appropriate category. Convolutional neural networks, which are commonly used for image classification tasks in pathology, may also be used for sentence classification (free-text pathology reports fit it this category). Other types of neural networks are also being used for text classification, such as long short-term memory (LSTM)^10^ and a more recent type of neural network called transformers.^2^

In this study, a convolutional neural network architecture was implemented to classify a set of cases into two categories: (1) Primary colonic adenocarcinoma. (2) Other cases that do not belong in the first category. After the model was trained, it was able to classify cases in the holdout set with an accuracy of 0.92 and AUC 0.957, which is acceptable for most real-world applications. The accuracy of 92 and AUC of .957 is expected to improve if more cases are added to the training set.

Although neural networks can be trained to be highly accurate in report classification, case searches using matching keywords is still a more viable option in many instances, especially when only a few cases are needed, or there are only a small number of cases available. This is due to the necessity of a large amount of labeled data for many neural networks, which can also be very time-consuming when thousands of data points are involved. Neural networks are less accurate when trained with a small dataset while also requiring additional steps for data preparation and analysis. Another drawback is the reduced interpretability of how the algorithm arrived at a particular result, where phrases are reduced to numbers and predictions are made based on a series of matrix calculations. Also, since this model was trained using data from one institution, its performance accuracy will probably be lower when it is applied to free-text pathology reports from other institutions due to the variability of how reports are phrased.

Improving the performance of neural networks commonly involves hyperparameter tuning and increasing the training set size. On the other hand, improving the performance of keyword-based searches involves selecting additional keywords to include or exclude cases of interest. For instance, false positives in this study included metastatic adenocarcinoma cases, results that contained “negative for carcinoma”, or cases from noncolonic regions. To reduce the number of false positives, cases containing “metastatic” or “negative for carcinoma” may be excluded, but this can also lead to missing cases of interest so the new keywords must be carefully chosen since a report containing “negative for carcinoma” may mention the presence of carcinoma in another section of the report.

Future considerations that could potentially improve model performance include the addition of other layers to the model, utilization of automated hyperparameter tuning methods and libraries, using other types of neural networks such as transformers and LSTM, as well as increasing the training set size. Other applications for NLP and neural networks in pathology include named entity recognition, disease categorization, and other types of classification problems.

## Conclusion

Trained CNN models by itself, or as an adjunct to keyword and pattern matching methods may be used to search for pathology cases of interest with high accuracy. Since manually labelling cases is a time-consuming endeavor, CNN assisted report identification is only recommended for large datasets, where the benefits may exceed the effort spent labeling a training set.

## Competing interests

There are no competing interests.

## References

[1] Bojanowski P., Grave E., Joulin A., Mikolov T. (2017). Enriching word vectors with subword information. TACL.

[2] Devlin J., Chang M.-W., Lee K., Toutanova K. (2019). BERT: pre-training of deep bidirectional transformers for language understanding. in: Proceedings of the 2019 Conference of the North American Chapter of the Association for Computational Linguistics: Human Language Technologies, Volume 1 (Long and Short Papers), Association for Computational Linguistics, Minneapolis, Minnesota.

[3] Mikolov T., Chen K., Corrado G., Dean J. (2013). Efficient estimation of word representations in vector space. arXiv.

[4] Trivedi H.M., Panahiazar M., Liang A. (2019). Large scale semi-automated labeling of routine free-text clinical records for deep learning. J Digit Imaging.

[5] Wang L., Wang Q., Bai H. (2020). EHR2Vec: representation learning of medical concepts from temporal patterns of clinical notes based on self-attention mechanism. Front Genet.

[6] Banerjee I., Chen M.C., Lungren M.P., Rubin D.L. (2018). Radiology report annotation using intelligent word embeddings: applied to multi-institutional chest CT cohort. J Biomed Inform.

[7] Campanella G., Hanna M.G., Geneslaw L. (2019). Clinical-grade computational pathology using weakly supervised deep learning on whole slide images. Nat Med.

[8] Bueno G., Fernandez-Carrobles M.M., Gonzalez-Lopez L., Deniz O. (2020). Glomerulosclerosis identification in whole slide images using semantic segmentation. Comput Methods Programs Biomed.

[9] Bentaieb A., Hamarneh G. (2018). Adversarial stain transfer for histopathology image analysis. IEEE Trans Med Imaging.

[10] Ye J.J. (2019). Construction and utilization of a neural network model to predict current procedural terminology codes from pathology report texts. J Pathol Inform.

